# Supplementary material for: The association between cigarette affordability and consumption: An update
Source: PLoS One. 2018 Dec 5;13(12):e0200665. doi: 10.1371/journal.pone.0200665 (PMC6281249; doi:10.1371/journal.pone.0200665)
Supplement: S1 Appendix — (DOCX) [file pone.0200665.s001.docx]

**Appendix 1: List of countries of each economy type included in Figure 1, 3, and 5**

| Low-income economies | Lower middle-income economies | Upper middle-income economies | High-income economies |
| --- | --- | --- | --- |
| Azerbaijan† | Algeria | Argentina† | Australia* |
| Bangladesh* | China† | Brazil | Austria |
| Cambodia* | Colombia | Chile† | Bahrain* |
| Cameroon | Ecuador | Costa Rica | Belgium |
| Cote d'Ivoire* | Egypt, Arab Rep. | Czech Republic | Canada |
| India | Guatemala | Hungary | Denmark |
| Indonesia | Iran, Islamic Rep. | Malaysia | Finland |
| Kenya | Morocco | Mexico | France |
| Nepal* | Paraguay* | Oman* | Germany |
| Nigeria* | Peru | Panama* | Greece |
| Pakistan | Philippines | Poland | Hong Kong SAR, China |
| Papua New Guinea* | Romania | Saudi Arabia | Iceland* |
| Senegal* | Russian Federation | Uruguay | Ireland |
| Ukraine | Serbia |  | Italy |
| Uzbekistan† | South Africa |  | Japan |
| Vietnam | Sri Lanka* |  | Korea, Rep. |
|  | Thailand |  | Kuwait* |
|  | Tunisia |  | Luxembourg* |
|  | Turkey |  | Netherlands |
|  |  |  | New Zealand* |
|  |  |  | Norway |
|  |  |  | Portugal |
|  |  |  | Qatar |
|  |  |  | Singapore |
|  |  |  | Spain |
|  |  |  | Sweden |
|  |  |  | Switzerland |
|  |  |  | United Arab Emirates† |
|  |  |  | United Kingdom |
|  |  |  | United States |
| 16 | 19 | 13 | 30 |

Note:

† denotes countries that were excluded from Figure 4 due to the missing of CPI

* denotes countries that were excluded from regression analysis due to the missing of consumption data.
