# Supplementary material for: The association between cigarette affordability and consumption: An update
Source: PLoS One. 2018 Dec 5;13(12):e0200665. doi: 10.1371/journal.pone.0200665 (PMC6281249; doi:10.1371/journal.pone.0200665)
Supplement: S2 Appendix — (DOCX) [file pone.0200665.s002.docx]

**Appendix 2: The effect of cigarette affordability on per capita ALL consumption**

| Control Variables | Specification (1) | | Specification (2) | |
| --- | --- | --- | --- | --- |
| Log of cigarette affordability 1 | -.194***  (.050) | -.174***  (.050) |  |  |
| Log of cigarette affordability 2 |  |  | -.175***  (.052) | -.168***  (.053) |
| Population aged 15-64, % of total | .084***  (.018) | .089***  (.018) | .107***  (.014) | .106***  (.015) |
| Population female, % of total | .070***  (.016) | .070***  (.016) | .102***  (.014) | .100***  (.015) |
| Unemployment, % of total labor force | .042**  (.015) | .042**  (.015) | .032**  (.011) | .033**  (.011) |
| Composite MPOWER Score |  |  | -.009  (.014) | -.004  (.016) |
| Year fixed effects | N | Y | N | Y |
| N | 887 | 887 | 519 | 519 |

Note:

Standard errors are clustered at country level.

Significance level: * p<0.05, ** p<0.01, *** p<0.001, † p<0.1.
